# Factors influencing vector control for onchocerciasis in Sub-Saharan Africa: A systematic review

**DOI:** 10.1371/journal.pntd.0014402

**Published:** 2026-08-14

**Authors:** Daniel N. Elakpa, Sumaya Mall, Juliana Kagura, Emmanuella Nzeribe, Latifat Ibisomi, Tobias Chirwa

**Affiliations:** 1 Faculty of Health Sciences, School of Public Health, University of the Witwatersrand, Johannesburg, South Africa; 2 Nigerian Institute of Medical Research, Abuja, Nigeria; 3 The Light Research and Consulting, Abuja, Nigeria; 4 BAMMISHO Health and Demographic Surveillance Systems Node, Rustenburg, South Africa; Cyprus International University: Uluslararasi Kibris Universitesi, CYPRUS

## Abstract

Onchocerciasis, commonly known as river blindness, remains a significant public health challenge in Sub-Saharan Africa, where the blackfly serves as its primary vector. This review synthesizes evidence on the factors influencing vector control programs in the region**.** A search was conducted in the Cochrane Library, PubMed, Web of Science, and Scopus to identify factors which influence the successful implementation of vector control programs in sub-Saharan Africa, covering studies published between 1 January 2000 and 31 March 2025, including both experimental and quasi-experimental designs. Study selection was conducted by two reviewers using Rayyan software, disagreements resolved by a third reviewer. Quality appraisal was assessed with JBI critical appraisal checklist. Findings were synthesized thematically and narrated. Seventeen studies were included in this review. The review identified several key determinants affecting vector control. Financial constraints were a major barrier, with high start-up and maintenance costs limiting the sustainability of larviciding programs. Community-based interventions, such as the ‘slash and clear’ method of removing trailing vegetation, were cost-effective but required consistent local commitment. Improper application of larvicides and the emergence of insecticide resistance posed additional challenges, as did environmental factors such as high rainfall and fast river currents, which affected breeding site accessibility and intervention efficacy. The success of vector control programs for onchocerciasis in Sub-Saharan Africa is influenced by several factors which requires enhanced investment, community engagement, and technical capacity building for the long-term success of onchocerciasis elimination efforts in the region.

## Author summary

Onchocerciasis, or river blindness, is a parasitic disease transmitted by the bites of infected blackflies (*Simulium* species). Control efforts in sub-Saharan Africa have historically relied on mass drug administration (MDA) of ivermectin, sometimes combined with vector control to reduce blackfly populations. While MDA is highly effective at reducing parasite loads in humans, persistent transmission can occur in areas with high biting rates, hard-to-reach breeding sites, or where drug coverage is suboptimal. We conducted a systematic review to identify factors associated with the success or failure of vector control interventions for onchocerciasis in sub-Saharan Africa. We examined evidence from aerial and ground larviciding, community-led “Slash and Clear,” Esperanza Window Traps, and other approaches. Our analysis revealed that ecological context, operational feasibility, seasonal timing, community engagement, and integration with ivermectin MDA are critical to success. These findings provide practical insights for programme planners, helping them choose and adapt vector control strategies based on local conditions. By targeting the right interventions to the right places, in combination with continued MDA, countries can accelerate progress toward the elimination of onchocerciasis.

## Introduction

Onchocerciasis (river blindness) remains a major public health concern in sub‑Saharan Africa, where over 120 million people are at risk and approximately 26 million are infected [1]. The disease causes chronic skin and eye morbidity, including severe dermatitis, visual impairment, and blindness [2], and has been linked to neurological complications, such as onchocerciasis-associated epilepsy [3–5]. The socioeconomic consequences such as reduced productivity, stigma, and long‑term poverty make it one of the most debilitating neglected tropical diseases [6,7].

Over the past four decades, regional initiatives such as the Onchocerciasis Control Programme (OCP) and the African Programme for Onchocerciasis Control (APOC) made substantial progress: OCP and APOC made substantial progress: OCP relied primarily on large-scale aerial larviciding, while APOC was centred on community-directed treatment with ivermectin (CDTI), with independent vector control deployed in only a limited number of settings [1,8–10]. Ivermectin‑based mass drug administration (MDA) has significantly reduced transmission in many settings [11]. However, persistent transmission continues in several foci particularly where biting rates remain high, or treatment coverage is suboptimal [12,13]. Evidence from Cameroon, the DRC, and other high‑transmission areas suggests that MDA alone may be insufficient to interrupt transmission, especially in hyperendemic or ecologically complex settings [14,15].

### Overview of main vector control strategies for onchocerciasis

Vector control for *Onchocerca volvulus* primarily involves aerial and ground larviciding with temephos, which targets *Simulium* larvae in fast‑flowing rivers. Aerial larviciding, developed under the OCP, allows treatment of large and inaccessible breeding sites, while ground larviciding is used in smaller river systems, with both requiring repeated applications due to reinfestation. The Slash and Clear method which is the removal of trailing vegetation along riverbanks is a low‑cost, community‑led approach effective in small, accessible rivers but less feasible in large or heavily vegetated systems. Esperanza Window Traps (EWTs), odour‑baited and insecticide‑treated, provide a targeted option for reducing adult blackfly biting, especially when breeding sites cannot be reached. Other supplementary measures include environmental management and integrated strategies that combine vector control with ivermectin MDA to reduce both vector density and human parasite reservoirs.

Beyond pharmacological interventions, vector control has demonstrated substantial potential in reducing onchocerciasis transmission. In the elimination era, renewed attention has focused on vector control, which historically achieved interruption of transmission in confined settings such as Bioko Island, yet remains underutilized across mainland Africa due to operational, financial, and environmental constraints [8,10]. Since 2016, the Expanded Special Project for the Elimination of Neglected Tropical Diseases (ESPEN) has supported countries to integrate such strategies into post‑APOC elimination efforts [16]. However, despite decades of MDA and localized vector control activities, persistent transmission in several foci highlights gaps in our understanding of the determinants of successful implementation. Building on earlier experiences such as the OCP’s demonstration that vector control alone can achieve long‑term interruption [17], there is a clear need to clarify which determinants influence program performance in diverse African settings.

To address this gap, this systematic review aims to synthesize existing evidence to identify and categorize the key barriers and facilitators affecting vector control outcomes for onchocerciasis in sub‑Saharan Africa. Therefore, the central guiding question of this review is: What factors are associated with the success or failure of vector control for onchocerciasis in Sub-Saharan Africa as reported in the existing literature?

## Methods

### Ethics statement

Ethics waiver was obtained from the University of the Witwatersrand Health Research Ethics Committee, with the number (REF: W-CBP-230324–01) as this study is a systematic review.

### Study design

This systematic review followed the Preferred Reporting Items for Systematic Reviews and Meta-Analyses (PRISMA 2020) guidelines [18]. The protocol was registered with PROSPERO on 28 November 2022 (CRD42022376792).

### Eligibility criteria

We included peer‑reviewed experimental and quasi‑experimental studies evaluating the implementation of vector control interventions for onchocerciasis in sub‑Saharan Africa between 1 January 2000 and 31 March 2025. Eligible studies assessed environmental, operational, biological, or chemical factors influencing the effectiveness of interventions targeting *Simulium* spp. Observational studies, case reports, conference abstracts, commentaries, reviews, and non‑English publications were excluded. We focused on experimental designs because they offer stronger attribution between interventions and measurable outcomes (e.g., changes in biting rates or infection indices). Excluding observational studies narrows contextual breadth but was necessary to ensure methodological comparability across included interventions. The chosen date range was selected to capture contemporary vector control strategies and emerging operational practices relevant to the 21st‑century elimination agenda. To maintain methodological rigor, we excluded studies unrelated to onchocerciasis control, as well as non‑English publications, case reports, conference abstracts, commentaries, observational studies, and reviews. The exclusion of non‑English literature reflects the authors’ language constraints; while this may limit completeness, it ensures accurate appraisal and interpretation of the included studies. (Full eligibility criteria and PICOST table, see Table 1 below.

**Table 1:** Eligibility criteria and PICOST.

P	Population	Blackflies in Sub-Saharan African countries
I	Intervention	Vector control methods
C	Comparator	N/A
O	Outcome	Vector control (observed in the control of onchocerciasis/number of disease cases as well as number of bites)
S	Studies	Experimental or Quasi-experimental design.
T	Timeline	2000-2025

### Search strategy

A comprehensive search was conducted in PubMed, Scopus, Web of Science, and the Cochrane Library. Search terms incorporated MeSH and Emtree headings and multiple synonyms for onchocerciasis, vector control, and *Simulium* spp. Search strategies for each database are provided in Tables A – D in S1 Appendix. The initial search covered February 1–March 1, 2023, with an updated search on March 6, 2025 to capture recent publications.

### Study screening and selection

Records were imported into Rayyan (rayyan.ai, a web-based systematic review management platform that facilitates collaborative screening and deduplication of references) for screening and duplicate removal. Two reviewers independently screened titles and abstracts against the eligibility criteria, followed by full‑text review of potentially relevant studies. Disagreements were resolved through discussion, and unresolved cases were adjudicated through a consensus involving a third reviewer. Screening procedures adhered to PRISMA standards [18].

### Data extraction and synthesis

Data were extracted independently by one reviewer into a standardized Microsoft Excel form and cross‑checked by a second reviewer. Extracted fields included study location, design, intervention type, entomological outcomes, implementation characteristics, and reported facilitators or barriers. We conducted a narrative synthesis following established guidelines [19,20]. This involved four main steps, first, a preliminary synthesis of findings from individual studies into broad descriptive categories. Second, we grouped and coded findings thematically to identify patterns across studies. Third, we explored relationships within and between studies, such as common contextual factors, vector ecology, or intervention outcomes. Finally, we assessed the robustness of the synthesis by considering the methodological quality and consistency of findings across studies. This approach allowed us to thematically organize and interpret the diverse findings from the selected studies for our systematic review.

### Quality appraisal

We assessed the methodological quality of all included studies using the Joanna Briggs Institute (JBI) Critical Appraisal Checklist for Quasi‑Experimental Studies [21,22]. Each study was independently appraised by two reviewers who scored all checklist items and documented justifications for each judgment. Any discrepancies in scoring or interpretation between the two reviewers were first discussed in a consensus meeting. If agreement could not be reached, a third senior reviewer acted as an adjudicator to resolve the disagreement and reach a final decision. This three‑step disagreement‑resolution process ensured consistency, minimized bias, and strengthened methodological rigor.

## Results

**Fig 1:**
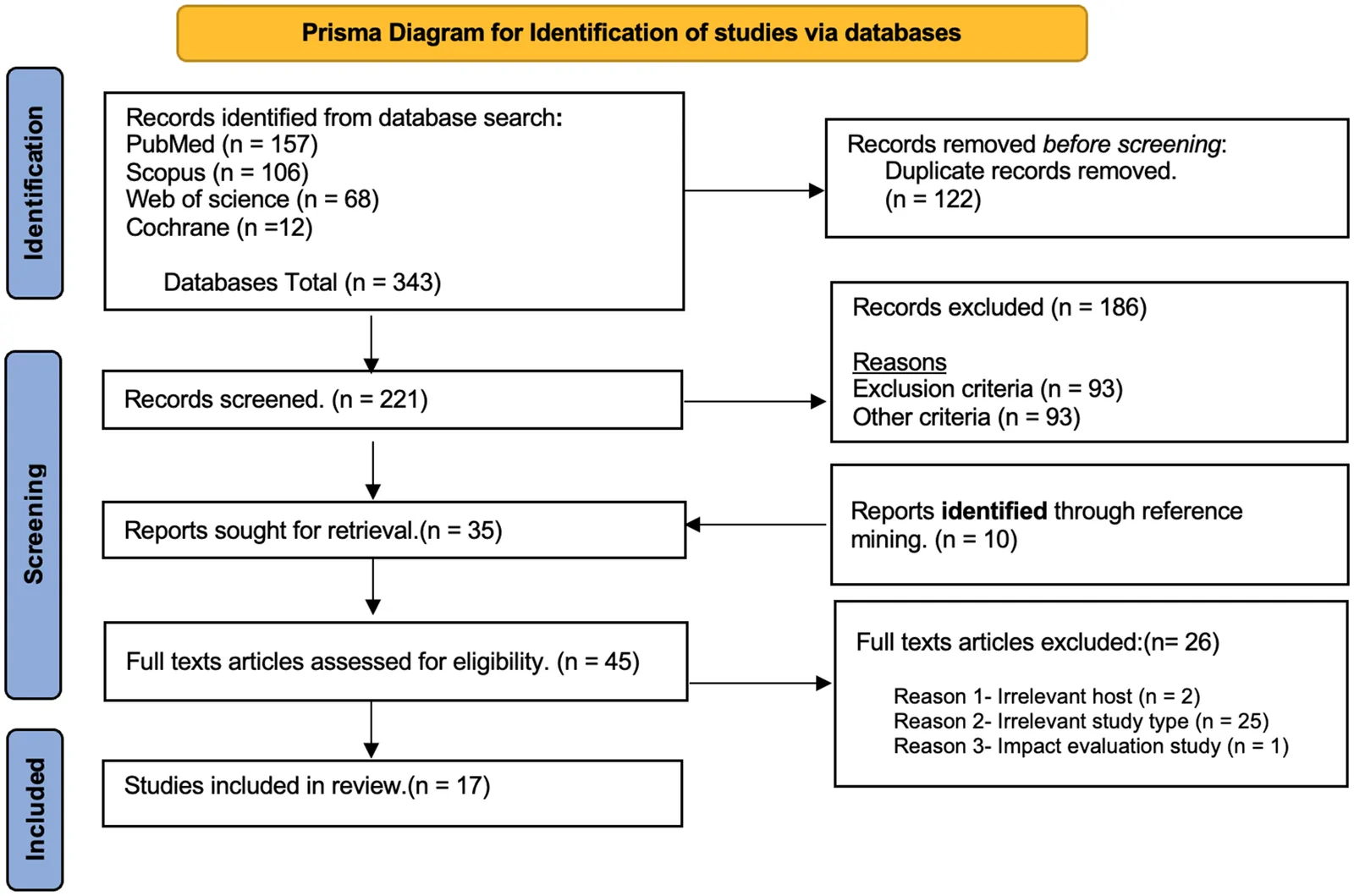
PRISMA diagram showing study selection process.

The systematic search yielded 343 records; 17 studies met the inclusion criteria. The results of the search strategy and the screening process are presented in the PRISMA diagram in Fig 1 to provide a clear overview. Table showing included can be found in Table 2 below. [Excluded studies from full text are presented in the in Table E in S1 Appendix]

**Table 2:** Table of included studies.

Study	Author/Country/ Year of Publication/Reference	Study Design	Summary of Findings	Factors Identified
Study 1	Jacob *et al.*, Uganda 2018	Quasi- experimental	Slash and Clear resulted in 89–99% declines in vector biting rates. The effect lasted up to 120 days post intervention.	Re-colonization of blackflies after treatment of breeding sites.
Study 2	Kalinga *et al.*, Tanzania 2007	Quasi- experimental	standard diagnostic dose of 1.25mg/l attained a mortality rate of 100%. The LC50 was from 0.129 – 0.34mg/l (95% CI, P < 0.05) After two years of intermittent treatment with temephos, larval mortality rate of 100% was 1.25mg/l	Blackfly Vector susceptibility to aerial larvicides (innate or acquired resistance due to exposure to agricultural insecticides). Incomplete vector elimination. Cross migration/re-colonization of treated breeding sites by new or residual. Populations migrating from nearby breeding areas.
Study 3	Loum *et al.*, Uganda 2019	Quasi- experimental	The mean fly collections per trap is 4-fold to 26-fold greater than HLC Deployment of the traps reduced the biting rate in classes by 91%. At the farm fields, each optimized trap collected on average 17 times more than the HLC. The optimization process resulted in a dramatic improvement in the performance of the EWT.”	When traps were used, the location is often important to determines effectiveness. Use of baits and types of baits. Political instability. Difference in species. No availability of trap design materials.
Study 4	Ekanya *et al.*, Cameroun 2022	Quasi- experimental	A mortality of 100% was recorded at concentrations between 0.5 and 0.1 mg/l. As the concentration of temephos decreased, mortality of larvae also decreased.	Ground larviciding with temephos was facilitated with the fact that the larvae are susceptible to temephos and this temephos was found not to be harmful to other aquatic fauna. The availability of fast-flowing rivers in this basin contributes to the large numbers of black files necessary for high transmission which may further require more larvicide that may end up harming the non-target aquatic fauna.
Study 5	Young *et al.*, Burkina Faso 2015	Quasi- experimental	The researchers identified 29 compounds common to the human sweat of three individuals. Several short chain carboxylic acids and aliphatic alcohols were stimulatory to *S. damnosum* s.l. and S. *ochraceum* s.l. respectively.	Technical and entomological capacity, variation among species of vector black flies.
Study 6	Zarroug *et al.*, Sudan 2019	Quasi- experimental	The dam complex has produced a positive effect by reducing the breeding sites of black flies in the area. Breeding sites of black flies were reduced due to construction of dams.	Dams alter the pattern of disease transmission, causing issues with public health.
Study 7	Routledge *et al.*, Ghana 2018	Quasi- experimental	The estimated efficacy of large-scale aerial larviciding in the savannah was greater than that of ground-based larviciding in the forest. Larviciding is more likely to succeed in areas with lower water temperatures and where blackfly species have longer gonotrophic cycles. At 93% larvicidal efficacy x 10 consecutive weekly larvicidal treatments would reduce DBRs by 96%. At 70% efficacy x 10 weekly applications, the DBR would decrease by 67%	Duration, frequency/interval of larviciding. River volume and flow; Temperatures; cytospecies identities, population dynamics and vector ecology. Larvicide efficacy or concentration. programmatic difficulties. Poor implementation process and inaccessible breeding sites. Seasonal feasibility of implementation, vector ecology, and the susceptibility of the aquatic stages of the local vector species
Study 8	Traoré *et al.*, Equatorial Guinea 2009	Quasi- experimental	The biting catches also showed a dramatic fall. At the eight regular capture sites no biting flies were collected or seen for the following 3 years.	Accessibility to the breeding sites. Re-colonization/re-invasion of vectors. inadequately treated breeding sites due to vegetation and insufficient insecticide.
Study 9	Lakwo *et al.*, Uganda 2006	Quasi- experimental	After river treatment, a drastic fall mean number of flies/hours from 3 flies/h in 2001–0 flies/h in 2003. The annual biting rates were reduced from 14 235 flies in 2001 to only 730 flies in 2003. There was also a reduction in the number of anthropophilic *Simulium* infection and infective rates.	Lack of useful data on transmission rates and elimination efforts may have prevented a comparison on the transmission rates prior to the river treatments. Prospects of success are higher in isolated regions (since there is no repopulation of vectors after treatment).
Study 10	Raimon *et al.*, Sudan 2021	Quasi- experimental	These monthly biting rates decreased drastically by 99% immediately after the “slash and clear” intervention. However, 2- months post-intervention, MBR spiked in all three sites. Although a rise in MBR was observed from the seventh month, it was not comparable to baseline figures.	The need to repeat the “slash and clear” intervention in the future every 7 months. Inadequate resources for costly larviciding. Lack of adequate entomological workforces. Local commitment and involvement.
Study 11	Garms *et al.*, Uganda 2009	Quasi- experimental	No immature stages were found on 494 crabs examined in January to March 1997. Vector assessment in February/March 2003 confirmed its interruption. No infested crabs have been found since then anywhere in the focus.	The concentration of the larvicide. Efficiency of the larviciding process.
Study 12	Crosa *et al.*, (A), Guinea 2001.	Quasi- experimental	No evidence of long-term trend in invertebrate community structures were present. There was evidence of medium-term variation for all 3 rivers and reflects seasonal changes in invertebrate densities. Low densities in results of invertebrate communities were identified after a short period.	Effect of larvicide on other biological organisms
Study 13	Crosa *et al.*, (B), Guinea 2001	Quasi- experimental	A long-term trend is seen, but the fluctuation mainly reflects the seasonal variation of invertebrate densities. There were also seasonal, flow-related variations, showing low values during the high flow periods and high values during the dry seasons.	hydrological and seasonal conditions. The two Guinean rivers were full and fast flowing rivers that contained a lot of fish, this facilitated the evaluation of fish densities before and after the intervention to see if the larviciding was harmful.
Study 14	Wilson *et al.*, Ghana 2012	Quasi- experimental	The catchers who used NO MAS caught (224 flies over 11 days - mean DBP of 20.4 flies per person. The duration of complete protection (time to first bite) achieved with NO MAS was 5 h (range = 5–7 h). The estimated overall protection achieved with NO MAS was 80.8%.	Actual protection will vary based on conditions such as temperature, perspiration and water exposure, and repellency may be compromised. Occupation of users influence the duration of repellency.
Study 15	Lakwo *et al.*, Uganda 2017	Quasi- experimental	Vector control trials using temephos revealed a reduction in crab infestation from 51% in July 2003 to 1.6% in February 2004.	Environmental factors (like deforestation and agriculture have led to the temporary disappearance of freshwater crabs. Type of vector species and intensity of transmission.
Study 16	Jacob *et al.*, Uganda 2021	Quasi- experimental	The biting rate was reduced by an average of 82% following the first treatment (slashing 0–1 km from a community) 21–42 days later. The bite rate was reduced by 99% overall after the second treatment (slashing 1–2 km away from the communities). Slashing the breeding sites within 0–2 km of the communities in all intervention communities decreased the mean daily biting rate to less than 1 bite/day. However, none of the intervention villages’ vector populations had fully recovered by the start of the subsequent rainy season.	vector control using insecticides is expensive and ecologically detrimental. Time of the season in which the (slash and clear) intervention is being conducted. i.e., seasonal variation of rainfall Entomological capacity is an important factor Willingness of community members and community ownership of programs Timing and frequency of slash and clear treatments Very large and dangerous rivers or in areas where the breeding sites are too small to be easily targeted by slash and clear
Study 17	Domche, *et al.*, Cameroon 2024	Quasi- experimental	Following the intervention, mean blackfly density in Biatsota declined by 50.8% (1936–953 flies) compared to a 26.7% reduction in the control village, Bayomen (2418–1774 flies), yielding an attributable intervention effect of 32.9% (p < 0.05). These findings demonstrate that Slash and Clear is a feasible and effective vector control method, even in high-transmission settings with large rivers and dense vector populations.	The intervention benefits from strong community participation and local ownership, which enhances sustainability. It targets breeding sites with trailing vegetation that are suitable for removal, using a low-cost, simple method requiring minimal technical equipment. This approach provides immediate efficacy in reducing vector density, particularly in high-transmission contexts. However, periodic repetition is necessary due to vegetation regrowth and potential recolonization from untreated sites. The accessibility of breeding sites influences the extent of coverage, while seasonal river flow and vegetation growth affect the optimal timing for intervention. Ultimately, the long-term success of the strategy depends on maintaining community motivation and engagement.

LC50 – Lethal Concentration 50; CI – Confidence interval; HLC -Human Landing Catch; EWT – Esperanza Window Trap; DBR – Daily Biting Rates; MBR – Mean Biting Rates; NO MAS (NM) – A brand of insect repellent.

### Evidence synthesis

Seventeen quasi-experimental studies were included in the review. Six of these studies were conducted in Uganda, two studies in each of Guinea, South Sudan, Cameroon, and Ghana, and only one study from each of Equatorial Guinea, Tanzania, and Burkina Faso. The studies included in the synthesis cover various aspects of onchocerciasis control, including vector control strategies, larviciding interventions, and the evaluation of different approaches.

Some of the studies included a preparatory phase before the actual study and data collection began. In terms of vector control methods, some of the studies explored the effectiveness of larviciding in reducing blackfly biting rates and transmission, while others explored slashing down trailing vegetations along fast flowing rivers. Furthermore, in this review, a number of studies focused on specific aspects related to vector ecology and behaviour, while another one study evaluated the effectiveness of the Esperanza Window Trap in reducing biting rates of *Simulium damnosum* sensu lato in Northern Uganda. In practice, the programmatic goal for much of Africa is not absolute vector elimination but reducing blackfly biting rates below the threshold necessary to sustain transmission, particularly when combined with regular ivermectin mass drug administration. Several interventions reviewed ranging from ground larviciding to community-led “Slash and Clear” approaches achieved substantial reductions in biting rates, thereby contributing to transmission suppression even in high-transmission settings.

Overall, the studies highlight the various factors which contribute to the success or failure of blackfly vector control programs. They include programmatic factors, vector-related factors (including ecology and biology), environmental factors, and human related factors such as technical (entomology) capacity or community involvement and commitment.

Programmatic factors refer to different elements essential for the successful and effective implementation of vector control programs. Four studies discussed programme related factors, one of these concerns is the duration of effect after a specific type of intervention such as ‘slash and clear’ [23–25]. If this is too short it can lead to a re-colonization of blackflies after treatment of breeding sites [1,26]. The effectiveness of Slash and Clear was facilitated by the presence of accessible trailing vegetation at breeding sites, which could be easily removed. However, the studies also highlighted that intervention feasibility is constrained by accessibility; inaccessible breeding sites remain potential sources of reinfestation. Seasonal fluctuations in river flow and vegetation growth further influence the optimal timing and sustainability of the intervention [23–25].

Biological and ecological factors drawn from characteristics of blackflies vectors and their interactions with the environment were identified by Four studies as important determinants of successful implementation of vector control programs [26–29]. For instance, blackfly vector susceptibility and resistance profiles to larvicides was mentioned in two studies as chief determinants to the success of larviciding control programs [26,28]. Additionally, understanding the blackfly habitat preference, the biting activity, and genetic mechanisms underlying insecticide resistance in blackflies is essential for developing effective strategies to combat resistance and tackle onchocerciasis. Other biological factors like natural predation and competition may have some consequences in the relative abundance of blackfly vectors [29].

Environmental factors were also found to have significant impact on the success or failure of vector control programs in Africa. Five studies discussed how environmental factors influence vector control programmes [24,30–33]. For example, seasonal variation of rainfall was identified as one important determinant of seasonal feasibility of vector control interventions This is because when the rainfall is high, it leads to large rivers bodies and fast flowing rivers which makes it difficult to reach breeding sites [24,28,30]. The presence of dams can also change the pattern of disease transmission by altering the speed and distribution of river flow, as well as creating varying pockets of breeding sites thereby creating more public health problems [29]. Isolation of regions or breeding sites such as islands can be considered an important criterion for achieving higher success.

Human related factors such as technical and entomological capacity of those involved in rolling out various vector control programs in Africa are part of the most important determinants of the success of vector control programs for blackfly, as highlighted in four studies from our review. [3,24,34,35] Entomological knowledge involves understanding blackfly’s ecological preference, their distribution and biting activity, knowing this will aid in the technical planning and preparatory phase for vector control programs as these could have significant implications [3,24,34,35]. For example, *Simulium damnosum* larvae more likely present in habitats near shrub [35]. On the other hand, local commitment, willingness of community members and community ownership of programs have been identified by several studies to be factors associated with vector control [3,24]. In Cameroon, while the immediate vector density reductions were notable, the study authors noted that vegetation regrowth and potential recolonization from untreated sites may require periodic repetition of the intervention [25]. They emphasized that, long-term success is dependent on sustained community motivation, ongoing resources, and the integration of slash and clear process into regular vector control cycles.

**Fig 2:**
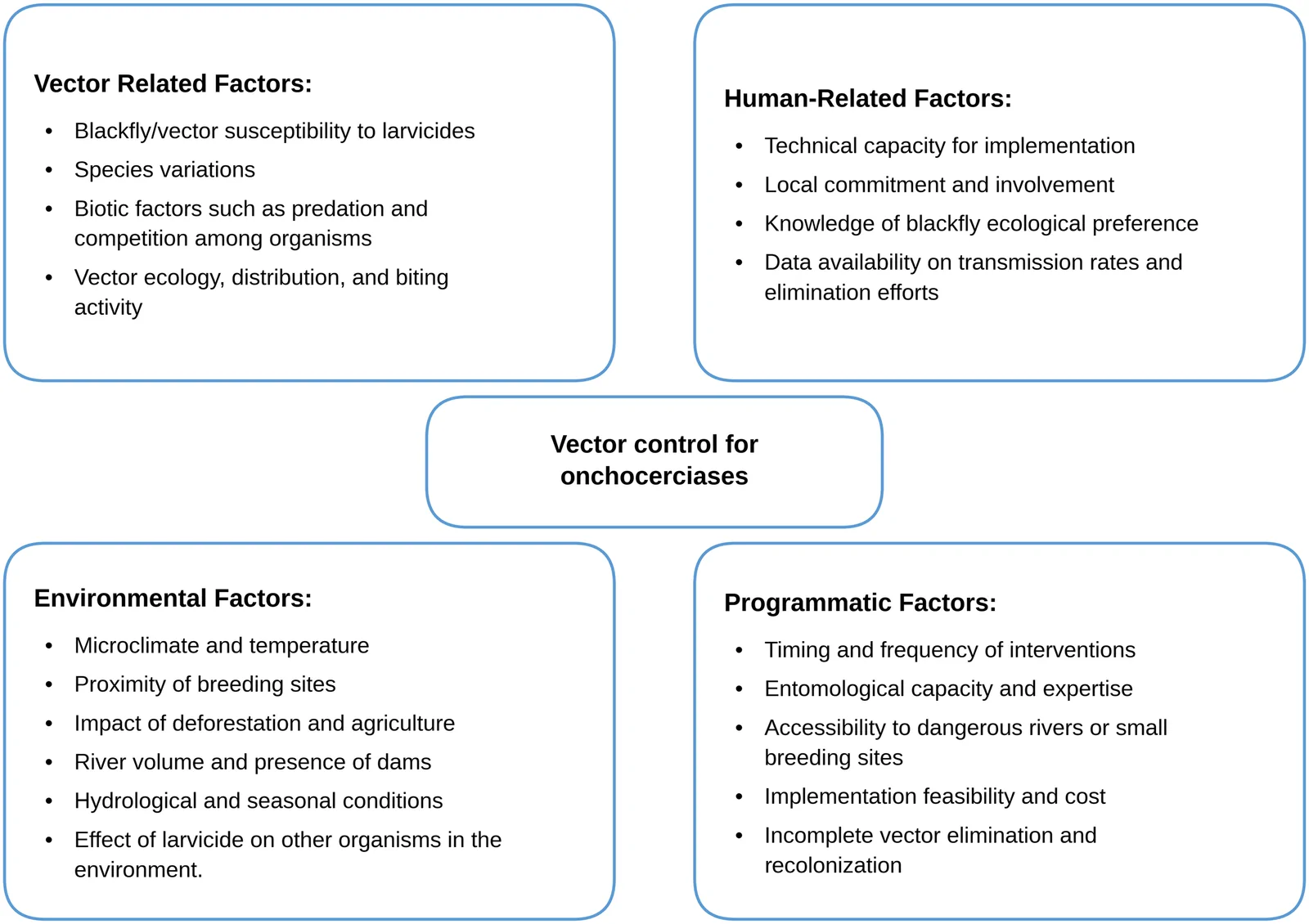
Conceptual diagram.

Other factors like presence of vegetation and shrubs, deforestation and agricultural activities have led to the temporary disappearance of freshwater crabs which are carriers to the blackfly larvae [1,26,36]. Political instabilities and insecurity have also hampered the sustainability and success of vector control activities in some regions [27]. These challenges disrupt operations, limit funding and resources, weaken health systems, hinder surveillance, and reporting, impede community engagement, and create coordination issues. The Fig 2 below shows a conceptual diagram with a snapshot of identified factors from our review.

### Quality appraisal

The JBI quality appraisal tools for quasi-experimental studies was employed for evaluating the quality of the included studies and gaining insights into the limitations of the evidence [22]. The quality appraisal table can be found in Table 3 below. All 17 included studies employed a quasi-experimental design. Overall quality scores, assessed using the Joanna Briggs Institute (JBI) critical appraisal checklist for quasi-experimental studies, ranged from 4 to 9, indicating variation in methodological rigor across the evidence base. Six studies (Studies 1, 2, 5, 7, 14, 17) scored 8–9, meeting most JBI criteria such as clear cause–effect attribution, appropriate comparison groups, valid and reliable outcome measures, and minimal risk of bias. These studies demonstrated a clear definition of cause and effect, with no confusion regarding the temporal relationships.

**Table 3:** Quality Appraisal.

Study	Study ID	Methodology/Study Design	Overall score
Study 1	Jacob *et al.,* Uganda 2018	Quasi- experimental	9
Study 2	Kalinga *et al.,* Tanzania 2007	Quasi- experimental	8
Study 3	Loum *et al.,* Uganda 2019	Quasi- experimental	7
Study 4	Ekanya *et al.,* Cameroun 2022	Quasi- experimental	4
Study 5	Young *et al.,* Burkina Faso 2015	Quasi- experimental	8
Study 6	Zarroug *et al.,* South Sudan 2019	Quasi- experimental	4
Study 7	Routledge *et al.,* Ghana 2018	Quasi- experimental	8
Study 8	Traoré *et al.,* Equatorial Guinea 2009	Quasi- experimental	5
Study 9	Lakwo *et al.,* Uganda 2006	Quasi- experimental	7
Study 10	Raimon *et al.,* South Sudan 2021	Quasi- experimental	7
Study 11	Garms *et al.*, Uganda 2009	Quasi- experimental	5
Study 12	Crosa *et al*., (A), Guinea 2001	Quasi- experimental	7
Study 13	Crosa *et al.,* (B), Guinea 2001	Quasi- experimental	6
Study 14	Wilson *et al.*, Ghana 2012	Quasi- experimental	9
Study 15	Lakwo *et al.*, Uganda 2017	Quasi- experimental	6
Study 16	Jacob *et al.*, Uganda 2021	Quasi- experimental	7
Study 17	Domche, *et al.*, Cameroon 2024	Quasi- experimental	8

They also involved pre- and post-measurements of the outcome in a reliable way and employed appropriate statistical analysis methods. Seven studies (Studies 3, 9, 10, 12, 13, 15, 16) scored 6–7, meeting several quality criteria but showing limitations in areas such as blinding, completeness of follow-up, or clarity in participant recruitment. These studies further strengthened their design by including control groups for comparison. Four studies (Studies 4, 6, 8, 11) scored 4–5, reflecting greater methodological limitations, including limited baseline comparability, absence of control groups, and incomplete reporting of outcomes. These studies also had issues with the definition and measurement of exposure and outcome variables, raising concerns about the validity and reliability of their findings. Although quality varied, lower-scoring studies contributed valuable contextual insights, particularly on operational feasibility and real-world implementation of vector control strategies in challenging field settings. The inclusion of multiple high-scoring studies strengthens confidence in the synthesis, while findings from lower-quality studies should be interpreted with caution. In general, since the majority of the included studies indicated a moderate to high level of quality, the implication is that the findings from this review are somewhat reliable.

## Discussion

This review synthesizes how programmatic, ecological, environmental, and human/institutional factors jointly shape the prospects for onchocerciasis vector control in sub‑Saharan Africa. Historical experience shows that sustained, well‑timed vector control can interrupt transmission, but only when it is operationally feasible, adequately financed, and embedded in strong surveillance systems. The Onchocerciasis Control Programme (OCP) demonstrated that repeated larviciding, executed at scale and synchronized with vector ecology, can eliminate vectors in suitable settings [1], in contrast, APOC extended coverage to countries outside the OCP zone but was built primarily around CDTI rather than vector control; independent vector control was used in only a limited number of settings, and cross-border reinfestation has been a long-standing challenge in post-OCP settings [9,16]. These lessons remain directly relevant to the current ESPEN era, where programmatic emphasis is on elimination and verification and where revitalized, context‑specific vector control can accelerate progress when coupled with high‑quality mass drug administration.

Based on the findings from our review, three implications emerge. First, vector control is most effective where breeding sites are identifiable and accessible and where sustained cycles of treatment can be maintained (e.g., Bioko Island’s elimination after persistent larviciding) [1]. Second, isolation matters: islands and geographically contained foci are less prone to reinvasion, while river basins spanning borders require coordinated schedules and shared data [8]. Third, relying solely on MDA may be insufficient in hyperendemic zones with high biting rates and complex breeding networks; targeted vector control remains a critical complement to reduce biting below transmission thresholds [10,13].

Across studies, operational feasibility (access to breeding sites, safe river conditions, and adequate frequency/duration of interventions) and implementation quality (correct concentrations, appropriate intervals) consistently distinguished success from failure [23,24]. Suboptimal larvicide dosing contributes to incomplete kill and fosters resistance; over‑treatment risks non‑target impacts and community backlash [32,33], conversely, overuse or excessive concentrations of larvicides pose ecological risks, including reduced aquatic biodiversity, toxicity to non‑target species, and long‑term environmental contamination. Chemicals may persist in aquatic systems, accumulate in the food chain, and cause bioaccumulation in higher organisms [37,38]. Where resources for aerial/ground larviciding are limited, Slash and Clear offers a low‑cost, community‑directed option, but its benefits wane with vegetation regrowth and require regular, planned repetition and community ownership to be sustainable.

Our review show that interventions must be species‑ and site‑informed. Cytospecies differences in gonotrophic cycles, habitat preferences, and susceptibility profiles influence larviciding schedules and the suitability of community‑led measures [29]. Seasonality (e.g., high rainfall and swift currents) can both hinder access and wash larvae downstream, complicating timing windows; dams alter hydrology and can create new breeding niches, requiring adaptive surveillance [31,39]. These dynamics shows why pre‑intervention entomology and routine monitoring are indispensable. In addition to these, we cannot rule out the impact of human and institutional determinants such as expertise, local leadership and community motivation [40]. Sustained gains depend on these, because shortages of trained personnel, turnover, and fragile operating environments impede continuity. Conversely, community‑directed models (e.g., Slash and Clear) perform best where ownership and safety protocols are strong, logistics are predictable, and feedback from entomological surveillance inform repeat cycles [3,24]. These dynamics are further illustrated by examples from the wider literature. In several Ugandan foci, *Simulium neavei* disappeared naturally from former transmission areas, a change likely linked to deforestation and the consequent loss of the freshwater crabs on which its larvae depend, demonstrating how human-driven environmental change can inadvertently alter vector ecology and transmission potential [36,41]. Conversely, the construction and subsequent abandonment of the Maridi Dam in South Sudan appears to have created favorable blackfly breeding conditions, contributing to a resurgence of *Onchocerca volvulus* transmission in the area [3,42].

Emerging challenges that reshape implementation of vector control includes insecticide resistance, reinfestation and reduced funding. These threats require careful consideration and renewed priority setting and planning. Insecticide resistance was documented to temephos in some blackfly control contexts from our review. This demands stewardship plans such as rotation, mosaic strategies, and routine susceptibility testing to mitigate ongoing and emergent development of resistance [26,43]. These challenges are complex, because in addition there are reported concerns that overuse or excessive concentrations of larvicides pose severe ecological risks to the environment, including reduced aquatic biodiversity, toxicity to non‑target species, and long‑term environmental contamination [44]. The problem of cross‑border reinfestation has been a recurrent weakness since APOC, this necessitates synchronized river‑basin operations, data sharing, and joint entomology teams across administrative boundaries. Furthermore, reduced donor support and competing priorities risk under‑funding of the very capacities (surveillance, training, logistics) that keep vector incidence low and prevent recrudescence; resilience requires domestic co‑financing and embedding vector control in health and environmental systems [10,16].

Evidence from this review supports Integrated Vector Management (IVM), matching interventions to local eco‑epidemiology and co‑optimizing with MDA. Practical combinations include community‑led Slash and Clear in small accessible rivers, ground/aerial larviciding for extensive or inaccessible habitats, and targeted traps (e.g., Esperanza Window Traps) to suppress biting between MDA rounds. Programs can now leverage GIS‑enabled mapping, mobile data collection, and remote sensing to prioritize reaches with persistent biting, schedule interventions to hydrological windows, and (iii) audit coverage and impact. Adoption of open, shared geo‑databases which are coordinated through ESPEN and national NTD units can reduce duplication and support cross‑border coordination [16,30].

This review has several limitations. First, restricting inclusion to English‑language publications may have introduced language bias and excluded relevant evidence from Francophone countries. Second, the heterogeneity of designs, settings, outcomes (e.g., DBR/MBR metrics, varying follow‑up), and intervention mixes precluded meta‑analysis; consequently, inferences rely on narrative synthesis. Third, although we included studies up to March 2025, relatively few recent quasi‑experimental publications were available, which may limit timeliness for rapidly evolving operational practices. Fourth, excluding observational/descriptive studies and most grey literature may omit valuable implementation lessons from routine programs and NGOs. Finally, while we applied the JBI checklist to assess study quality, we did not perform a formal certainty‑of‑evidence grading across outcomes.

Based on the findings of this review, the following policy and operational recommendations are proposed to accelerate the elimination agenda through ESPEN and country-specific national programmes. Programmes should first optimise CDTI coverage and compliance before escalating to more resource-intensive vector control interventions; mathematical modelling tools such as EPIONCHO-IBM can help assess whether CDTI alone is sufficient to interrupt transmission in a given focus. Where vector control is additionally indicated, the SIMPOP blackfly population model can guide larviciding schedules by simulating efficacy under site-specific ecological and hydrological conditions. Institutionalize resistance management: routine susceptibility testing; rotate/mosaic larvicides; maintain Quality Assurance/Quality Control on dosing; and document non‑target impacts with environmental monitoring. Adopt river‑basin, cross‑border compacts: synchronized treatment calendars, shared GIS layers of breeding sites, and joint entomological teams to mitigate reinvasion. Scale community‑directed models where feasible (small rivers), ensuring safety training, PPE, refresh cycles aligned to vegetation regrowth, and micro‑incentives that sustain participation. Digitize surveillance: standard mobile forms for biting/DBR/MBR, geotagged larvicide applications, and near‑real‑time dashboards for course correction. Invest in entomological capacity: multi‑year workforce plans, mentorship schemes, and regional reference labs to support species ID, resistance testing, and Quality Assurance. To reduce dependence on volatile external funding, programs should: Establish multi‑year domestic budget lines for vector surveillance and operations within NTD programs. Mobilize co‑financing from sectors that benefit directly from reduced biting/nuisance (e.g., hydropower, irrigation, tourism) and integrate vector control within environmental and social safeguard plans for dams and water projects.

## Conclusion

This review has identified several key factors associated with vector control for onchocerciasis in Sub-Saharan Africa. The elimination agenda in Africa will move faster where programs re‑integrate vector control with MDA using IVM, time operations to hydrology and species ecology, and invest in surveillance, resistance management, and cross‑border coordination. With pragmatic financing and digital tools, countries can drive biting below transmission thresholds, shorten time‑to‑interruption, and protect gains during the post‑MDA surveillance phase.

## Supporting information

S1 Appendix Table A. Search Strategy for PubMed. Table B. Search strategy for Scopus. Table C. Search strategy for Cochrane. Table D. Search strategy for Web of Science. Table E. of Excluded Studies.
(DOCX)

S1 PRISMA Checklist From: Page MJ, McKenzie JE, Bossuyt PM, Boutron I, Hoffmann TC, Mulrow CD, et al. The PRISMA 2020 statement: an updated guideline for reporting systematic reviews. BMJ 2021;372:n71. https://doi.org/10.1136/bmj.n71. (PDF)
