# Supplementary material for: Factors influencing vector control for onchocerciasis in Sub-Saharan Africa: A systematic review
Source: PLoS Negl Trop Dis. 2026 Aug 14;20(8):e0014402. doi: 10.1371/journal.pntd.0014402 (PMC13502913; doi:10.1371/journal.pntd.0014402)
Supplement: Supplementary file 2 [file pntd.0014402.s001.docx]

APPENDIX 1: Search Strategy

Table A Search Strategy for PubMed.

| Search number | Query |
| --- | --- |
| 1 | (onchocercias* OR river blindness OR onchocercal disease) AND (Vector manag* OR vector control) AND (Sub-saharan africa OR Africa) AND onchocerca volvulus AND (*simulium sirbanum* OR *simulium damnosum*) |
| 2 | (onchocercias* OR river blindness OR onchocercal disease) AND (Vector manag* OR vector control) AND (Sub-saharan africa OR Africa) AND onchocerca volvulus AND (*simulium sirbanum* OR *simulium damnosum*) |
| 3 | (((("Onchocerciasis"[Mesh] OR "Onchocerciasis, Ocular"[Mesh]) AND "Insect Control"[Mesh]) AND "Africa South of the Sahara"[Mesh]) AND "Onchocerca volvulus"[Mesh]) AND "Simuliidae"[Mesh] |
| 4 | (((("Onchocerciasis"[Mesh] OR "Onchocerciasis, Ocular"[Mesh]) AND "Insect Control"[Mesh]) AND "Africa South of the Sahara"[Mesh]) AND "Onchocerca volvulus"[Mesh]) AND "Simuliidae"[Mesh] |
| 5 | (((("Onchocerciasis"[Mesh] OR "Onchocerciasis, Ocular"[Mesh]) AND "Insect Control"[Mesh]) AND "Africa"[Mesh]) AND "*Onchocerca volvulus*"[Mesh]) AND "Simuliidae"[Mesh] |
| 6 | "Onchocerciasis"[Mesh] OR "Onchocerciasis, Ocular"[Mesh] OR "Insect Control"[Mesh] OR "Africa South of the Sahara"[Mesh] OR "Africa"[Mesh] OR "Onchocerca volvulus"[Mesh] OR "Simuliidae"[Mesh] |
| 7 | ((onchocercias* OR river blindness OR onchocercal disease) AND (Vector manag* OR vector control) AND (Sub-saharan africa OR Africa) AND onchocerca volvulus AND (*simulium sirbanum* OR *simulium damnosum*)) AND ((onchocercias* OR river blindness OR onchocercal disease) AND (Vector manag* OR vector control) AND (Sub-saharan africa OR Africa) AND *onchocerca volvulus* AND (*simulium sirbanum* OR *simulium damnosum*)) |
| 8 | ((onchocercias* OR river blindness OR onchocercal disease) AND (Vector manag* OR vector control) AND (Sub-saharan africa OR Africa) AND onchocerca volvulus AND (*simulium sirbanum* OR *simulium damnosum*)) AND ((onchocercias* OR river blindness OR onchocercal disease) AND (Vector manag* OR vector control) AND (Sub-saharan africa OR Africa) AND onchocerca volvulus AND *(simulium sirbanum* OR *simulium damnosum)*) AND ((((("Onchocerciasis"[Mesh] OR "Onchocerciasis, Ocular"[Mesh]) AND "Insect Control"[Mesh]) AND "Africa South of the Sahara"[Mesh]) AND "Onchocerca volvulus"[Mesh]) AND "Simuliidae"[Mesh]) AND ((((("Onchocerciasis"[Mesh] OR "Onchocerciasis, Ocular"[Mesh]) AND "Insect Control"[Mesh]) AND "Africa South of the Sahara"[Mesh]) AND "Onchocerca volvulus"[Mesh]) AND "Simuliidae"[Mesh]) AND ((((("Onchocerciasis"[Mesh] OR "Onchocerciasis, Ocular"[Mesh]) AND "Insect Control"[Mesh]) AND "Africa"[Mesh]) AND "Onchocerca volvulus"[Mesh]) AND "Simuliidae"[Mesh]) AND ((((("Onchocerciasis"[Mesh] OR "Onchocerciasis, Ocular"[Mesh]) AND "Insect Control"[Mesh]) AND "Africa"[Mesh]) AND "Onchocerca volvulus"[Mesh]) AND "Simuliidae"[Mesh]) |
| 9 | (onchocercias*[Title/Abstract] OR onchocercal skin disease[Title/Abstract] OR river blindness[Title/Abstract] OR onchocercal disease[Title/Abstract] OR onchocerca volvulus[Title/Abstract] OR simulium sirbanum[Title/Abstract] OR simulium damnosum[Title/Abstract]) AND (Vector manag*[Title/Abstract] OR vector control[Title/Abstract]) AND (Africa[Title/Abstract]) |
| 10 | #8 AND #9 |
| 11 | #1 AND #9 |
| 12 | #2 AND #9 |
| 13 | #3 AND #9 |

Table B Search strategy for Scopus

| 1 | ( ( TITLE ( africa ) OR ABS ( africa ) ) AND PUBYEAR > 1999 AND PUBYEAR < 2025 ) AND ( ( TITLE ( "vector manag*" OR "vector control" ) OR ABS ( "vector manag*" OR "vector control" ) ) AND PUBYEAR > 1999 AND PUBYEAR < 2025 ) AND ( ( TITLE ( ( onchocercias* OR "onchocercal skin disease" OR "river blindness" OR "onchocercal disease" OR "onchocerca volvulus" OR "simulium sirbanum" OR "simulium damnosum" ) ) OR ABS ( ( onchocercias* OR "onchocercal skin disease" OR "river blindness" OR "onchocercal disease" OR "onchocerca volvulus" OR "simulium sirbanum" OR "simulium damnosum" ) ) ) AND PUBYEAR > 1999 AND PUBYEAR < 2025 ) | 54 |
| --- | --- | --- |

Table C Search strategy for Cochrane

| 1 | ((onchocercias* OR “onchocercal skin disease” OR “river blindness” OR “onchocercal disease” OR “onchocerca volvulus” OR “simulium sirbanum” OR “simulium damnosum”)):ab,ti |  |
| --- | --- | --- |
| 2 | (Vector NEXT manag* OR “vector control”):ab,ti |  |
| 3 | (Africa):ab,ti |  |
| 4 | #1 AND #2 AND #3 | 1 |
| 5 | MeSH descriptor: [Onchocerciasis] explode all trees |  |
| 6 | MeSH descriptor: [Africa] explode all trees |  |
| 7 | #5 OR #6 |  |
| 8 | #4 and #7 | 1 |
| 9 | 2000-present | 0 |

Table D Search strategy for Web of Science

| ID | Search | Hits |
| --- | --- | --- |
| #1 | MeSH descriptor: [undefined] explode all trees | 0 |
| #2 | [**Error**]==> (((("Onchocerciasis"[Mesh] OR "Onchocerciasis, Ocular"[Mesh]) AND "Insect Control"[Mesh]) AND "Africa South of the Sahara"[Mesh]) AND "Onchocerca volvulus"[Mesh]) AND "Simuliidae"[Mesh] |  |
| #3 | MeSH descriptor: [Onchocerciasis, Ocular] explode all trees | 21 |
| #4 | MeSH descriptor: [Onchocerciasis] explode all trees | 156 |
| #5 | MeSH descriptor: [Insect Control] explode all trees | 268 |
| #6 | MeSH descriptor: [Africa South of the Sahara] explode all trees | 7571 |
| #7 | MeSH descriptor: [Onchocerca] explode all trees | 75 |
| #8 | MeSH descriptor: [Simuliidae] explode all trees | 6 |
| #9 | #3 OR #4 AND #5 AND #6 AND #7 AND #8 | 22 |
| #10 | ((onchocercias* OR river blindness OR onchocercal disease) AND (Vector manag* OR vector control) AND (Sub-saharan africa OR Africa) AND onchocerca volvulus AND (*simulium sirbanum* OR *simulium damnosum*)):ti,ab,kw (Word variations have been searched) | 1 |
| #11 | ((onchocercias* OR river blindness OR onchocercal disease) AND (Vector manag* OR vector control) AND (Sub-saharan africa OR Africa) AND onchocerca volvulus AND (*simulium sirbanum* OR *simulium damnosum*)(onchocercias* OR river blindness OR onchocercal disease) AND (Vector manag* OR vector control) AND (Sub-saharan africa OR Africa) AND onchocerca volvulus AND (*simulium sirbanum* OR *simulium damnosum*)):ti,ab,kw (Word variations have been searched) | 0 |
| #12 | (onchocercias* OR river blindness OR onchocercal disease) AND (Vector manag* OR vector control) AND (Sub-saharan africa OR Africa) AND onchocerca volvulus AND (*simulium sirbanum* OR *simulium damnosum*) | 0 |
| #13 | (onchocercias* OR “onchocercal skin disease” OR “river blindness” OR “onchocercal disease” OR “onchocerca volvulus” OR “simulium sirbanum” OR “simulium damnosum”) (Title) or (onchocercias* OR “onchocercal skin disease” OR “river blindness” OR “onchocercal disease” OR “onchocerca volvulus” OR “simulium sirbanum” OR “simulium damnosum”) (Abstract) |  |
| #14 | “Vector manag*” OR “vector control” (Title) or “Vector manag*” OR “vector control” (Abstract) |  |
| #15 | (Africa) (Title) or (Africa) (Abstract) | 49 |

Table E of Excluded Studies

| STUDY | REASON FOR EXCLUSION |
| --- | --- |
| Long-term, large-scale biomonitoring Of the unknown: Assessing the Effects of Insecticides to Control River Blindness  (Onchocerciasis) in West Africa. (1) | No study design. No results/outcomes were reported. |
| Habitat preference of blackflies in Omo Gibe river basin (southwest Ethiopia): Implications for onchocerciasis elimination and control. (35) ***[Main reference list]*** | This study has an observational design |
| Accelerating river blindness elimination by supplementing MDA with a vegetation “slash and clear” vector control strategy: a data driven modeling analysis.(2) | This article was a data driven modelling analysis, had no study design. |
| Seasonal Variation in Biting Rates of Simulium damnosum sensu lato, Vector of Onchocerca volvulus, in Two Sudanese Foci.(3) | This study has an observational design |
| Vector control and entomological capacity for onchocerciasis elimination. (40) ***[Main reference list]*** | A review assessing achievements and pitfalls of vector control interventions and has no study design |
| Longitudinal evaluation of repellent activity of Ocimum gratissimum (Labiatae) volatile oil against *Simulium damnosum.*(4) | An evaluation study and did not focus on the scope of our research question. |
| Historical Elimination of Onchocerciasis from Victoria Nile Focus in Central Uganda Verified Using WHO Criteria.(5) | A routine program-monitoring activity (evaluating the historical effectiveness of vector control from 1974) |
| Factors affecting onchocerciasis transmission: lessons for infection control.(6) | This article is a discussion piece/expert commentary |
| Bacteriome Diversity of Blackflies’ Gut and Association with Onchocerca volvulus, the Causative Agent of Onchocerciasis in Mbam Valley (Center Region, Cameroon).(7) | Focused on bacteriomes of blackflies and does not answer our research question. |
| Refocusing vector assessment towards the elimination of onchocerciasis from Africa: a review of the current status in selected countries.(8) | A review and has no primary data nor study design. |
| Implementation of test‑and‑treat  with doxycycline and temephos ground  larviciding as alternative strategies  for accelerating onchocerciasis elimination  in an area of loiasis co‑endemicity:  the COUNTDOWN consortium multi‑disciplinary  study protocol.(9) | A protocol for ongoing research, thus has no primary data to answer our research question |
| ‘Slash and clear’ vector control for onchocerciasis elimination and epilepsy prevention: a protocol of a cluster randomised trial in Cameroonian villages. (4) ***[Main reference list]*** | A protocol for ongoing research, thus has no primary data to answer our research question |
| The impact of ivermectin treatment alone and when in parallel with *Simulium neavei* elimination on onchocerciasis in Uganda.(11) | This study was comparing effectiveness and did not answer our research question |
| An 18S ribosomal DNA barcode for the study of Isomermis lairdi, a parasite of the blackfly *Simulium damnosum* s.l. (12) | Focused on exploring a parasite's potential role in reducing blackfly vector population densities, thus not relevant to our study. |
| Onchocerca volvulus infection and serological prevalence, ocular onchocerciasis and parasite transmission in northern and central Togo after decades of *Simulium damnosum* s.l. vector control and mass drug administration of ivermectin (13) | This was an assessment study for a post -elimination + MDA program and not relevant to our review |
| Onchocerciasis in West Africa after 2002: a challenge to take up.(14) | This article was a discussion piece. Does not meet our inclusion criteria, thus irrelevant to our study |
| Control of onchocerciasis (15) | This article was a discussion piece. Does not meet our inclusion criteria, thus irrelevant to our study |
| Review of the National Program for Onchocerciasis Control in the Democratic Republic of Congo.(16) | This study was a review. Does not meet our inclusion criteria, thus irrelevant to our study |
| The geographic distribution of onchocerciasis in the 20 participating countries of the African Programme for Onchocerciasis Control: (1) priority areas for ivermectin treatment.(17) | This was a mapping study. Does not meet our inclusion criteria, thus irrelevant to our study |
| Deforestation and the spatio-temporal distribution of savannah and forest members of the *Simulium damnosum* complex in southern Ghana and south-western Togo.(18) | This was a mapping study. Not relevant to our study/wrong type of study |
| Potential effects of warmer worms and vectors on onchocerciasis transmission in West Africa.(19) | This was a mapping and modelling study. Not relevant to our study/wrong type of study |
| Population Fluctuations and Effect of Climatic Factors on the Relative Abundance of *Simulium damnosum* Complex (Diptera: Simuliidae)(20) | This study was an assessment study and not focused on vector control. Not relevant to our study/wrong type of study |
| Onchocerciasis Elimination: Progress and Challenges.(21) | This study was a review of the current status the disease. Not relevant to our study/wrong type of study |
| Identification of Human-Derived Attractants to *Simulium damnosum* Sensu Stricto in the Madi-Mid North Onchocerciasis Focus of Uganda.(22) | Outcomes not relevant to our study |
| Indices of onchocerciasis transmission by different members of the *Simulium damnosum* complex conflict with the paradigm of forest and savanna parasite strains.(23) | Outcomes not relevant to our study/wrong type of study |
| Integrated rapid mapping of onchocerciasis and loiasis in the Democratic Republic of Congo: impact on control strategies. (By Tekle et al) | This is a mapping study and impact evaluation |
| Identification of the onchocerciasis vector in the Kakoi-Koda focus of the Democratic Republic of Congo.(24) | Outcomes not relevant to our study |
| Onchocerciasis control in the Democratic Republic of Congo (DRC): challenges in a post-war environment.(25) (By Bof et al) | Outcomes not relevant to our study |

# **References**

1.	Resh VH, Leveque C, Statzner B. Long-term, large-scale biomonitoring of the unknown: assessing the effects of insecticides to control river blindness (onchocerciasis) in West Africa. Annu Rev Entomol. 2004;49:115-39.

2.	Smith ME, Bilal S, Lakwo TL, Habomugisha P, Tukahebwa E, Byamukama E, et al. Accelerating river blindness elimination by supplementing MDA with a vegetation “slash and clear” vector control strategy: a data-driven modeling analysis. Scientific Reports. 2019;9(1).

3.	Zarroug IMA, Hashim K, Elaagip AH, Samy AM, Frah EA, ElMubarak WA, et al. Seasonal Variation in Biting Rates of Simulium damnosum sensu lato, Vector of Onchocerca volvulus, in Two Sudanese Foci. PLOS ONE. 2016;11(3):e0150309-e.

4.	Usip L, Opara KN, Ibanga ES, Atting IA. Longitudinal evaluation of repellent activity of Ocimum gratissimum (Labiatae) volatile oil against Simulium damnosum. Memorias do Instituto Oswaldo Cruz. 2006;101(2):201-5.

5.	Katabarwa MN, Habomugisha P, Khainza A, Oguttu DW, Byamukama E, Katamanywa J, et al. Historical Elimination of Onchocerciasis from Victoria Nile Focus in Central Uganda Verified Using WHO Criteria. The American Journal of Tropical Medicine and Hygiene. 2020;102(6):1411-.

6.	Cheke RA. Factors affecting onchocerciasis transmission: lessons for infection control. Expert Review of Anti-Infective Therapy: Taylor and Francis Ltd; 2017. p. 377-86.

7.	Efon Ekangouo A, Nana Djeunga HC, Sempere G, Kamgno J, Njiokou F, Moundipa Fewou P, et al. Bacteriome Diversity of Blackflies' Gut and Association with Onchocerca volvulus, the Causative Agent of Onchocerciasis in Mbam Valley (Center Region, Cameroon). Pathogens. 2021;11(1).

8.	Boakye D, Tallant J, Adjami A, Moussa S, Tekle A, Robalo M, et al. Refocusing vector assessment towards the elimination of onchocerciasis from Africa: A review of the current status in selected countries. International Health. 2018;10:i27-i32.

9.	Wanji S, Nji TM, Hamill L, Dean L, Ozano K, Njouendou AJ, et al. Implementation of test-and-treat with doxycycline and temephos ground larviciding as alternative strategies for accelerating onchocerciasis elimination in an area of loiasis co-endemicity: The COUNTDOWN consortium multi-disciplinary study protocol. Parasites and Vectors. 2019;12(1).

10.	Siewe Fodjo JN, Vieri MK, Ngarka L, Njamnshi WY, Nfor LN, Mengnjo MK, et al. Slash and clear' vector control for onchocerciasis elimination and epilepsy prevention: A protocol of a cluster randomised trial in Cameroonian villages. BMJ Open. 2021;11(9).

11.	Ndyomugyenyi R, Tukesiga E, Büttner DW, Garms R. The impact of ivermectin treatment alone and when in parallel with Simulium neavei elimination on onchocerciasis in Uganda. Tropical Medicine and International Health. 2004;9(8):882-6.

12.	Crainey JL, Wilson MD, Post RJ. An 18S ribosomal DNA barcode for the study of Isomermis lairdi, a parasite of the blackfly Simulium damnosum s.l. Med Vet Entomol. 2009;23(3):238-44.

13.	Komlan K, Vossberg PS, Gantin RG, Solim T, Korbmacher F, Banla M, et al. Onchocerca volvulus infection and serological prevalence, ocular onchocerciasis and parasite transmission in northern and central Togo after decades of Simulium damnosum s.l. vector control and mass drug administration of ivermectin. PLoS Neglected Tropical Diseases. 2018;12(3).

14.	Hougard JM, Yameogo L, Philippon B. Onchocerciasis in West Africa after 2002: a challenge to take up. Parasite. 2002;9(2):105-11.

15.	Boatin B. The Onchocerciasis Control Programme in West Africa (OCP). Annals of Tropical Medicine and Parasitology. 2008;102(SUPPL. 1).

16.	Makenga Bof JC, Ntumba Tshitoka F, Muteba D, Mansiangi P, Coppieters Y. Review of the National Program for Onchocerciasis Control in the Democratic Republic of the Congo. Trop Med Infect Dis. 2019;4(2).

17.	Noma M, Zoure HG, Tekle AH, Enyong PA, Nwoke BE, Remme JH. The geographic distribution of onchocerciasis in the 20 participating countries of the African Programme for Onchocerciasis Control: (1) priority areas for ivermectin treatment. Parasit Vectors. 2014;7:325.

18.	Wilson MD, Cheke RA, Flasse SP, Grist S, Osei-Ateweneboana MY, Tetteh-Kumah A, et al. Deforestation and the spatio-temporal distribution of savannah and forest members of the Simulium damnosum complex in southern Ghana and south-western Togo. Trans R Soc Trop Med Hyg. 2002;96(6):632-9.

19.	Cheke RA, Basanez MG, Perry M, White MT, Garms R, Obuobie E, et al. Potential effects of warmer worms and vectors on onchocerciasis transmission in West Africa. Philos Trans R Soc Lond B Biol Sci. 2015;370(1665).

20.	Oforka LC, Adeleke MA, Anikwe JC, Makanjuola WA. Population Fluctuations and Effect of Climatic Factors on the Relative Abundance of Simulium damnosum Complex (Diptera: Simuliidae). Environ Entomol. 2019;48(2):284-90.

21.	Lakwo T, Oguttu D, Ukety T, Post R, Bakajika D. Onchocerciasis Elimination: Progress and Challenges. Res Rep Trop Med. 2020;11:81-95.

22.	Cozart D, Lakwo T, Liu C, Loum D, Jacob B, Cupp EW, et al. Identification of Human-Derived Attractants to Simulium damnosum Sensu Stricto in the Madi-Mid North Onchocerciasis Focus of Uganda. Am J Trop Med Hyg. 2020;103(4):1563-8.

23.	Cheke RA, Garms R. Indices of onchocerciasis transmission by different members of the Simulium damnosum complex conflict with the paradigm of forest and savanna parasite strains. Acta Trop. 2013;125(1):43-52.

24.	Post RJ, Laudisoit A, Mandro M, Lakwo T, Laemmer C, Pfarr K, et al. Identification of the onchocerciasis vector in the Kakoi-Koda focus of the Democratic Republic of Congo. PLoS Negl Trop Dis. 2022;16(11):e0010684.

25.	Makenga Bof JC, Maketa V, Bakajika DK, Ntumba F, Mpunga D, Murdoch ME, et al. Onchocerciasis control in the Democratic Republic of Congo (DRC): challenges in a post-war environment. Tropical Medicine & International Health. 2015;20(1):48-62.
